# Supplementary material for: Longitudinal dynamics of gut bacteriome, mycobiome and virome after fecal microbiota transplantation in graft-versus-host disease
Source: Nat Commun. 2021 Jan 4;12:65. doi: 10.1038/s41467-020-20240-x (PMC7782528; doi:10.1038/s41467-020-20240-x)
Supplement: Supplementary file 1 — Supplementary Information [file 41467_2020_20240_MOESM1_ESM.pdf]

Longitudinal dynamics of gut bacteriome, mycobiome and virome after fecal microbiota transplantation in graft-versus-host disease

Supplementary Information

Supplementary Figures 1-11

Supplementary Table 1-4

a

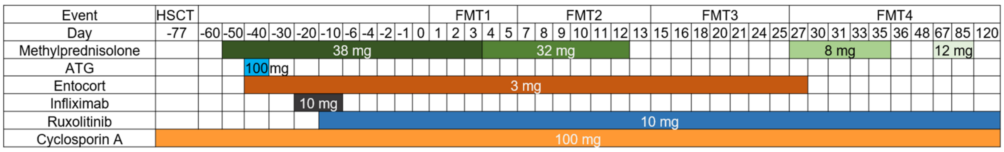

b

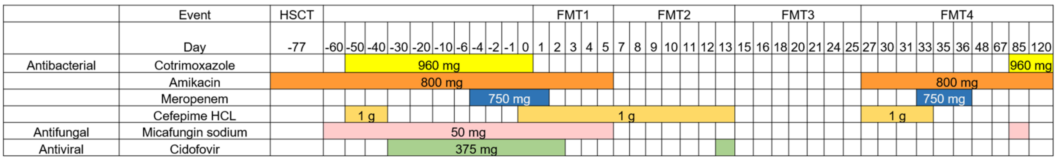

Supplementary Figure 1. Medical therapies the patient received during study follow-up. a. Immunosuppressants. b. Antibacterial, antifungal and antivirals. The dose represented intake per day.

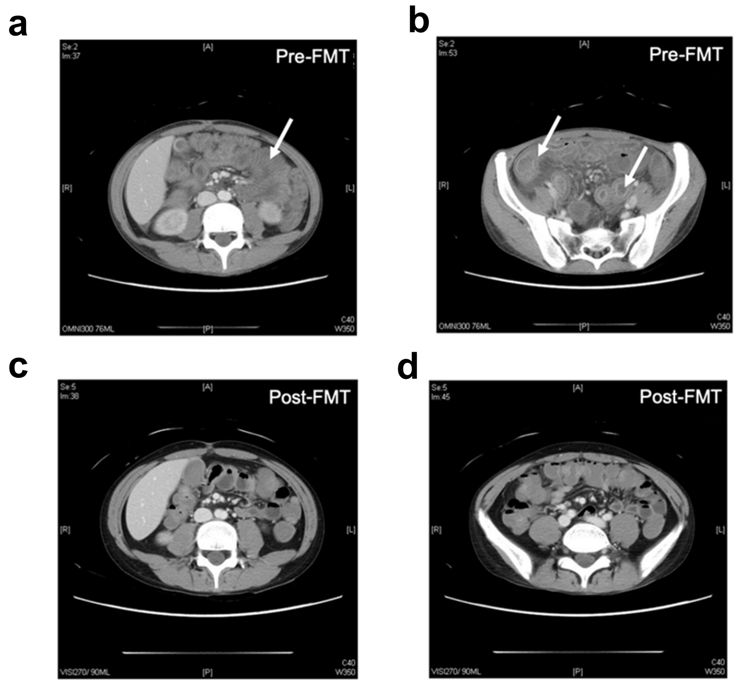

Supplementary Figure 2. Computed tomography (CT) scan images of patient before and after FMT. a, b. Abdominal and pelvic CT scan before FMT show diffuse inflammatory change and markedly thickened jejunum and distal ileum (white arrows). c, d. Abdominal and pelvic CT scan after fourth FMT show resolution of thickening of small bowel. The CT scan were performed for one time.

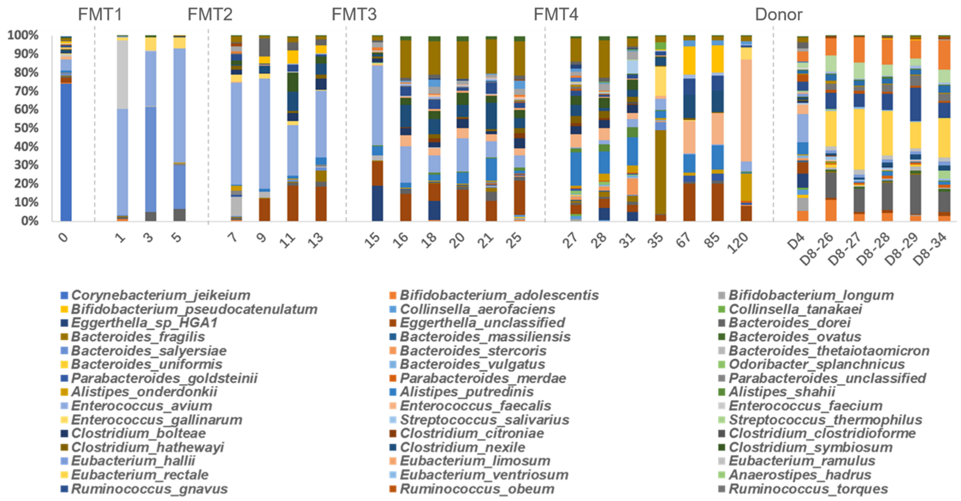

Supplementary Figure 3. Taxonomic structure of fecal bacterial microbiota at the species level based on metagenomics profiling.

Day 0 represents stool sample collected before the 1st FMT. FMT1, FMT2, FMT3, FMT4 represent stool samples collected after the 1st (day 1-5), 2nd (day 7-13), 3rd (day 15-25) and 4th (day 27-120) FMT respectively. D8-26, D8-27, D8-28, D8-29, D8-34 indicate fecal samples collected from donor D8 on different days, whereas an only single stool from D4 was used in the FMT. FMT1, FMT2, FMT3, FMT4 were performed using D8-26 and D8-27, D8-28 and D8-29, D8-34, D4 respectively.

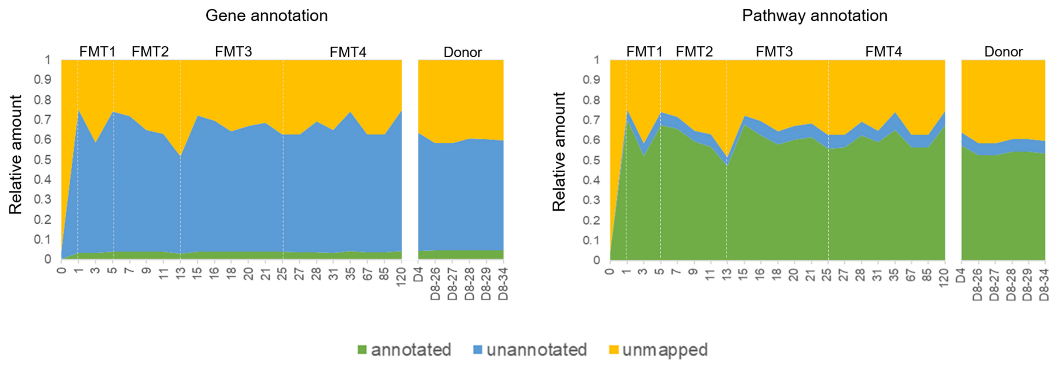

Supplementary Figure 4. Proportion of annotated, unannotated and unmapped reads in gene family (a) and pathway (b) in patient and donor's fecal samples. Gene family and pathway annotations were implemented by *HUMAnN2*. D8-26, D8-27, D8-28, D8-29 and D8-34 represent fecal samples from donor D8 collected on different days and D4 represents fecal sample from another donor D4 at a single time point. Day 0 represents stool sample collected before the 1st FMT, while 1-120 represented the days after the 1st FMT.

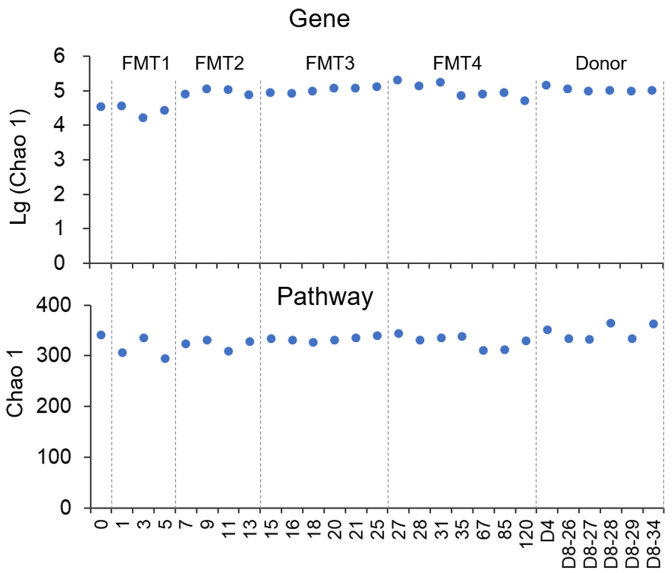

Supplementary Figure 5. Richness plots of gene families and pathways in donor stools and the patient. The richness was estimated by Chao1 index. D8-26, D8-27, D8-28, D8-29 and D8-34 represent fecal samples from donor D8 collected on different days and D4 represents fecal sample from another donor D4 at a single time point. Day 0 represents stool sample collected before the 1st FMT, while 1-120 represented the days after the 1st FMT.

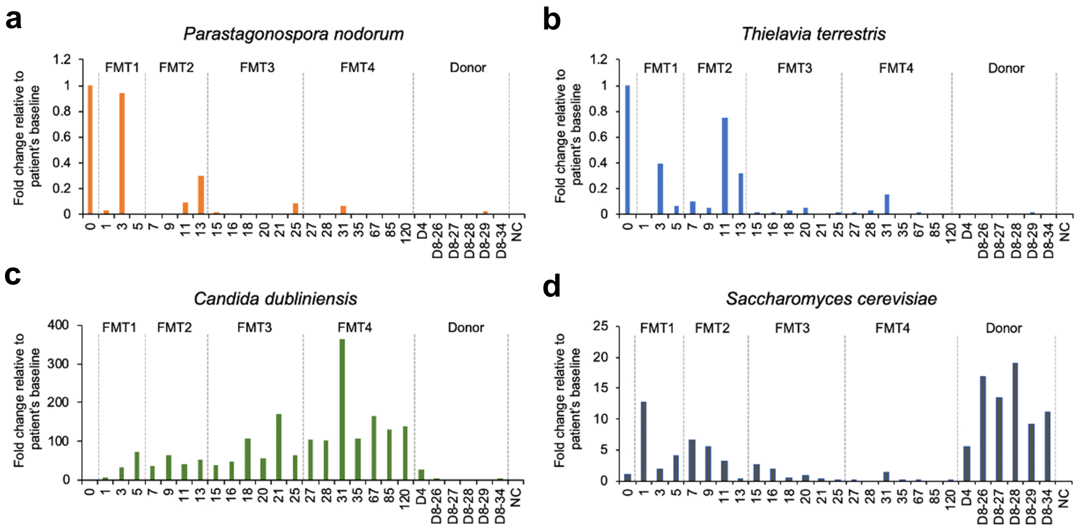

Supplementary Figure 6. Changes in abundance of fungal species, *Parastagonospora nodorum*, *Thielavia terrestris*, *Candida dubliniensis* and *Saccharomyces cerevisiae* after FMTs based on quantitative PCR analysis. Day 0 represents stool sample collected before the 1st FMT, while 1-120 represented the days after the 1st FMT. The changes over time were expressed as a fold change from day 0. D8-26, D8-27, D8-28, D8-29 and D8-34 represent fecal samples from donor D8 collected on different days and D4 represents fecal sample from another donor D4 at a single time point. NC indicates a negative control during DNA extraction.

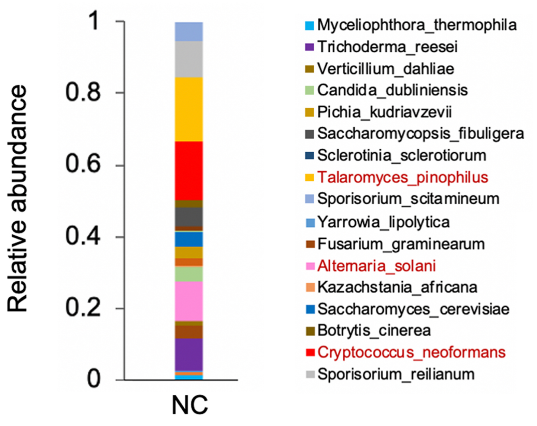

Supplementary Figure 7. Fungal composition in the sequenced negative control sample at species level. Negative control sample was sequenced when we performed DNA extraction. The most abundant species were highlighted in red color. One biologically independent sample examined over one independent experiment.

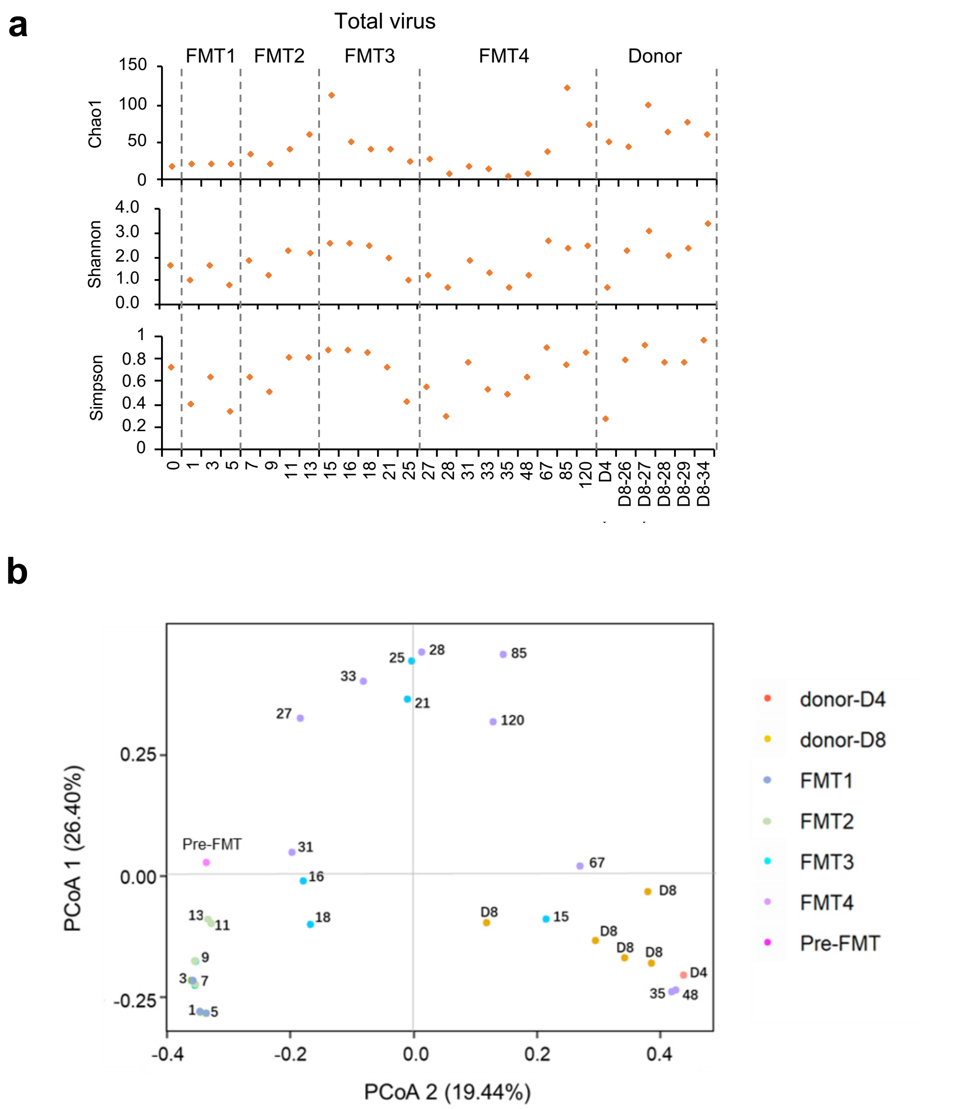

Supplementary Figure 8. Alpha diversity and Principal coordinate analysis (PCoA) of total viral community in donors and the patient before and after FMT. a. Richness, Shannon and Simpson diversity of whole virus communities in the patient as well as donors. b. PCoA of Bray-Curtis dissimilarities between fecal viral structures of the patient following FMTs. Pre-FMT represents stool sample collected before the 1st FMT. FMT1, FMT2, FMT3, FMT4 represent stool samples collected after the 1st (day 1-5), 2nd (day 7-13), 3rd (day 15-25) and 4th (day 27-120) FMT respectively.

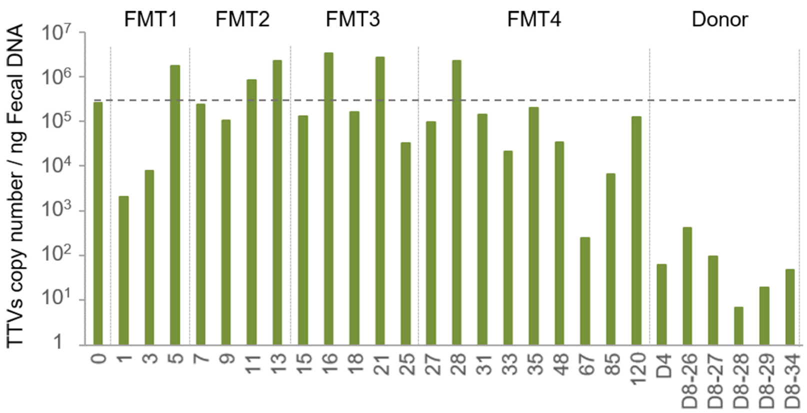

Supplementary Figure 9. Absolute fecal *Torque teno* virus levels in donor, patient, pre-FMT and post-FMT based on quantitative PCR analysis. Day 0 represents stool sample collected before the 1st FMT. FMT1, FMT2, FMT3, FMT4 represent stool samples collected after the 1st (day 1-5), 2nd (day 7-13), 3rd (day 15-25) and 4th (day 27-120) FMT respectively. D8-26, D8-27, D8-28, D8-29, D8-34 indicate fecal samples collected from donor D8 on different days, whereas an only single stool from D4 was used in the FMT. FMT1, FMT2, FMT3, FMT4 were performed using D8-26 and D8-27, D8-28 and D8-29, D8-34, D4 respectively.

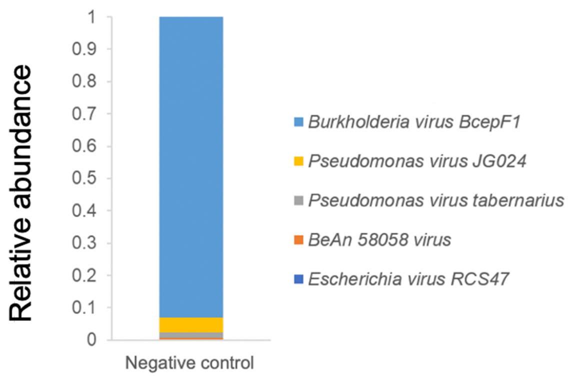

Supplementary Figure 10. Viral composition in the sequenced negative control sample at species level. Negative control sample (reagent control) was sequenced when we performed VLP extraction and amplification. One biologically independent sample examined over two independent experiments.

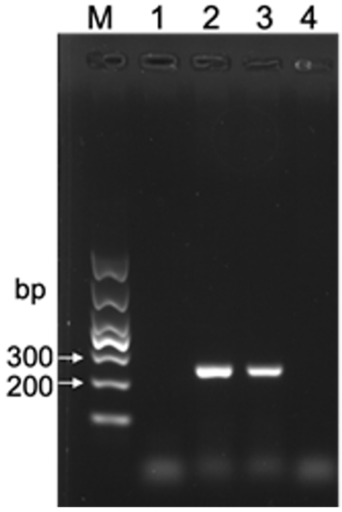

Supplementary Figure 11. PCR for detection of diphtheria toxin gene in patient's fecal DNA sample before FMT. Lane 1: Fecal DNA in patient before FMT; Lane 2: Positive control, diphtheria toxin produced strain, *C. diphtheriae* ATCC 13812; Lane 3, Positive control, diphtheria toxin produced strain, *C. diphtheriae* ATCC 19409; Lane 4: Negative control, dd H_20. The experiment was repeated for three times independently with similar results.

Supplementary Table 1 Clinical symptoms of patient after FMT

Day*	Stool times	Stool volume / mL	Vomit times	Vomit volume / mL	Stool form
#0	5	465	4	465	Reddish watery stool
1	1	100	10	330	Blood clot
2	3	294	3	294	Reddish brownish loose stool
3	1	110	5	156	Bloody loose stool
4	3	379	No record	No record	Old blood clot in stool
#5	2	293	2	293	Old blood loose to watery stool
7	1	52	2	127	Bloody thick stool
8	2	133	8	192	Old bloody stool
9	3	210	10	285	Brownish watery stool
10	1	91	14	340	Brownish watery stool
11	1	185	5	139	No record
12	1	119	1	154	Dark red bloody watery stool
#13	1	111	4	91	Dark red bloody watery stool
15	1	41	3	49	Dark brown old blood clot
16	2	126	2	235	Dark brown old blood clot
18	1	22	1	129	Old clot
20	1	29	1	58	No record
21	1	21	1	41	Dark red stool
24	0	0	1	45	NA
#25	1	121	2	40	Dark Red
27	1	120	1	50	Brick Red with clot
28	0	0	1	27	No record
30	1	No record	10	159	Dark red loose stool
31	1	157	15	210	Bricked red loose stool
33	1	300	6	58	Dark red loose stool
35	1	229	7	140	Dark red loose stool
36	3	673	3	68	Dark red loose stool
48	2	56	3	96	No record
67	1	55	2	76	Brownish watery stool
85	1	No record	1	7	No record
120	0	0	10	465	No record

#Patient received FMT on day 0, day 5, day 13 and day 25.

Supplementary Table 2. Read counts and statistics of fungi-enriched DNA metagenomes.

Sample Name	Clean Reads	Clean bases(G)	Q30(%)	GC(%)
0	92,173,894	13.8	97.44%	41.65%
1	72,443,758	10.9	97.92%	39.45%
3	88,204,656	13.2	97.51%	42.36%
5	73,588,520	11.0	97.79%	40.52%
7	77,765,468	11.7	97.78%	43.29%
9	70,532,626	10.6	97.88%	44.71%
11	67,385,372	10.1	97.76%	48.21%
13	58,134,470	8.7	97.60%	46.73%
15	65,234,924	9.8	97.64%	47.07%
16	59,517,262	8.9	97.67%	47.30%
18	62,048,846	9.3	97.65%	48.94%
20	65,415,230	9.8	97.71%	48.50%
21	63,318,344	9.5	97.45%	48.04%
25	63,537,426	9.5	97.66%	49.12%
27	65,411,708	9.8	97.61%	48.54%
28	65,204,608	9.9	97.56%	50.03%
31	64,447,868	9.7	97.67%	50.21%
35	63,337,152	9.5	97.58%	43.20%
48	65,267,400	9.8	97.77%	45.89%
67	58,533,746	9.8	97.71%	48.06%
85	61,624,666	9.2	97.62%	48.47%
120	59,848,414	8.9	97.72%	46.18%
D4	63,751,754	9.6	97.77%	46.82%
D8-26	59,776,440	9.0	97.86%	43.21%
D8-27	63,320,394	9.5	97.66%	43.80%
D8-28	60,163,854	9.0	97.82%	44.19%
D8-29	62,573,292	9.4	97.74%	43.01%
D8-30	72,198,190	10.8	97.88%	43.81%

Supplementary Table 3. Read counts and statistics of metagenomes enriched for virus-like particles (VLPs).

Sample	Clean reads	Clean bases(G)	Q30(%)	GC（%)
0	29,716,416.00	9	96.58	51.96
1	31,253,714.00	9.4	97.51	37.63
3	26,575,043.00	8	97.39	36.98
5	28,975,968.00	8.7	97.66	37.79
7	30,459,732.00	9.1	97.79	37.23
9	24,389,112.00	7.3	97.81	37.24
11	28,878,516.00	8.2	95.39	39.81
13	22,155,822.00	6.6	97.70	37.37
15	23,687,214.00	7.1	92.26	40.64
16	27,052,065.00	8.7	95.62	42.07
18	15,030,047.00	7.8	97.63	37.87
21	25,427,680.00	7.6	93.19	38.54
25	25,859,799.00	7.8	92.46	37.17
27	27,827,674.00	8.3	97.86	39.07
28	26,520,747.00	8	97.89	38.87
31	28,391,965.00	8.5	97.41	43.13
33	28,793,720.00	8.6	97.32	40.17
35	25,094,512.00	7.5	93.27	39.11
48	26,018,704.00	7.8	92.14	37.39
67	28,702,891.00	8.1	97.61	44.14
85	29,006,486.00	8.7	97.11	39.00
120	28,966,619.00	8.6	97.81	39.04
D4	26,306,413.00	7.9	93.63	40.45
D8-26	22,102,687.00	8.7	97.92	38.77
D8-27	22,336,033.00	6.7	97.31	40.25
D8-28	26,172,446.00	7.9	97.97	40.25
D8-29	27,592,303.00	6.6	97.98	39.39
D8-34	30,013,740.00	8.3	97.95	40.68

Supplementary Table 4. Sequence of the primers, probes and target genes.

Species	Primer or probe	Sequence (5′-3′)	Reference	Target gene[a] and primer position
Thielavia terrestris	*Tte-F*	GACCTTACCTAA CCGTTGCT	–	MK461104.1 (60-174)
	Tte-R	GCGAGAATGGT TTGGAGCCT		
Parastagonospora nodorum	*Pno-F*	TCTGCTTGGTG TTGGGTGTT	–	MN313349.1 (456-584)
	Pno-R	GCTTGTGGACG CAAGTGTTT		
Candida dubliniensis	*Cdu-F*	AACGCAGCGAA ATGCGATAC	–	MK394123.1 (1678-1800)
	Cdu-R	GAGGGAGAAAC GACGCTCAA		
S.cerevisiae	*SI4-F*	ATTGCTGGCCT TTTCATTG	39	MN622826.1 (539-701)
	SI7-R	CGCCTAGACGC TCTCTTCTTAT		
Total fungi	*FungiQuant-F*	GGRAAACTCAC CAGGTCCAG	40	MH793495.1 (612-792)
	FungiQuant-R	GSWCTATCCCC AKCACGA		
	FungiQuant-P	(6FAM)5′-TGGTGCATGGC CGTT-3′ (MGBNFQ)		

F: forward primer; R: reverse primer; P: probe.

[a] the accession number of target genes were shown in this table.
