# Supplementary material for: Longitudinal dynamics of gut bacteriome, mycobiome and virome after fecal microbiota transplantation in graft-versus-host disease
Source: Nat Commun. 2021 Jan 4;12:65. doi: 10.1038/s41467-020-20240-x (PMC7782528; doi:10.1038/s41467-020-20240-x)
Supplement: Supplementary file 3 — Reporting Summary [file 41467_2020_20240_MOESM3_ESM.pdf]

nature research

Corresponding author(s): Siew C Ng and Paul KS Chan

Last updated by author(s): Oct 13, 2020

Reporting Summary

Nature Research wishes to improve the reproducibility of the work that we publish. This form provides structure for consistency and transparency in reporting. For further information on Nature Research policies, see Authors & Referees and the Editorial Policy Checklist .

Statistics

For all statistical analyses, confirm that the following items are present in the figure legend, table legend, main text, or Methods section.

n/a	Confirmed	
☐	☒	The exact sample size (*n*) for each experimental group/condition, given as a discrete number and unit of measurement
☐	☒	A statement on whether measurements were taken from distinct samples or whether the same sample was measured repeatedly
☐	☒	The statistical test(s) used AND whether they are one- or two-sided<br>*Only common tests should be described solely by name; describe more complex techniques in the Methods section.*
☒	☐	A description of all covariates tested
☒	☐	A description of any assumptions or corrections, such as tests of normality and adjustment for multiple comparisons
☒	☐	A full description of the statistical parameters including central tendency (e.g. means) or other basic estimates (e.g. regression coefficient) AND variation (e.g. standard deviation) or associated estimates of uncertainty (e.g. confidence intervals)
☒	☐	For null hypothesis testing, the test statistic (e.g. *F*, *t*, *r*) with confidence intervals, effect sizes, degrees of freedom and *P* value noted<br>*Give P values as exact values whenever suitable.*
☒	☐	For Bayesian analysis, information on the choice of priors and Markov chain Monte Carlo settings
☒	☐	For hierarchical and complex designs, identification of the appropriate level for tests and full reporting of outcomes
☐	☒	Estimates of effect sizes (e.g. Cohen's *d*, Pearson's *r*), indicating how they were calculated

Our web collection on statistics for biologists contains articles on many of the points above.

Software and code

Policy information about availability of computer code

Data collection	Trimmomatic v0.36, Kneaddata v0.73, humann2 v0.11.1, BMTagger v3.101-1, Bowtie 2 v2.3.5, Kraken 2 v2.07-beta, DIAMOND v2.0.4, MetaPhlAn2 v2.96.1-0, HumanMycobiomeScan, QIIME2 v2018.6
Data analysis	R v3.5.1, phyloseq v1.26.0, vegan v2.5-3, pheatmap v1.0.10

For manuscripts utilizing custom algorithms or software that are central to the research but not yet described in published literature, software must be made available to editors/reviewers. We strongly encourage code deposition in a community repository (e.g. GitHub). See the Nature Research guidelines for submitting code & software for further information.

Data

Policy information about availability of data

All manuscripts must include a data availability statement. This statement should provide the following information, where applicable:

- Accession codes, unique identifiers, or web links for publicly available datasets
- A list of figures that have associated raw data
- A description of any restrictions on data availability

16S rRNA gene sequencing data is available in the NCBI Sequence Read Archive under BioProject PRJNA515137 (https://www.ncbi.nlm.nih.gov/bioproject/PRJNA515137). Metagenomic sequence data generated from fungi-enriched fecal DNA and virus like particles (VLPs)-enriched fecal viral DNA for this study are available in the NCBI Sequence Read Archive under BioProject accession PRJNA641975 (https://www.ncbi.nlm.nih.gov/bioproject/PRJNA641975).

The public reference databases used in this study are as follows: GRCh38 p12 (https://www.ncbi.nlm.nih.gov/assembly/GCF_000001405.38/), ChocoPhlAn database (http://huttenhower.sph.harvard.edu/humann2_data/chocophlan/chocophlan.tar.gz), UniRef90 universal protein reference database (https://www.uniprot.org/help/uniref), Metacyc database (https://metacyc.org/), Fungal database from HumanMycobiomeScan (https://sourceforge.net/projects/hmscan/), The complete NCBI viral RefSeq databases (https://www.ncbi.nlm.nih.gov/refseq/).

Field-specific reporting

Please select the one below that is the best fit for your research. If you are not sure, read the appropriate sections before making your selection.

☒ Life sciences ☐ Behavioural & social sciences ☐ Ecological, evolutionary & environmental sciences

For a reference copy of the document with all sections, see nature.com/documents/nr-reporting-summary-flat.pdf

Life sciences study design

All studies must disclose on these points even when the disclosure is negative.

Sample size	There is one single case GvHD patient study.
Data exclusions	No data was excluded
Replication	For the GvHD patient receiving 4 doses of FMT treatment, thirty-one stool samples from different time points were sequentially collected and analyzed. For donors D4 and D8, one stool sample from D4 and five from D8 were analyzed. For each stool specimen, one single sampling was conducted and subjected to analysis,
Randomization	The patient was firstly randomized to a antibiotic treatment (control treatment) to which the patient did not respond, folllowed by an serial fecal microbiota transplantaion treatment (experimental treatment) after which the symptoms of patients were alleviated .
Blinding	Physicians who evaluated patient symptoms during follow-up were not aware of the treatment being administered. Physicians who prepared the fecal infusions and conducted FMT did not assess the treatment outcome or performed the analysis. The authors who performed the microbiome analysis were initially made blind to the treatment followed by unblinding after completion of microbiome data extraction, upon which correlation and data extrapolation were made based on patient's treatment data and metadata.

Reporting for specific materials, systems and methods

We require information from authors about some types of materials, experimental systems and methods used in many studies. Here, indicate whether each material, system or method listed is relevant to your study. If you are not sure if a list item applies to your research, read the appropriate section before selecting a response.

Materials & experimental systems

n/a	Involved in the study	
☒	☐	Antibodies
☒	☐	Eukaryotic cell lines
☒	☐	Palaeontology
☒	☐	Animals and other organisms
☐	☒	Human research participants
☐	☒	Clinical data

Methods

n/a	Involved in the study	
☒	☐	ChIP-seq
☒	☐	Flow cytometry
☒	☐	MRI-based neuroimaging

Human research participants

Policy information about studies involving human research participants

Population characteristics	The patient is a 14-year-old male suffering from myelodysplastic syndrome with monosomy 7 underwent HLA-identical sibling allo-HSCT. The patient developed stage II skin GvHD (rash 25-50%), stage IV gut GvHD with profuse bloody diarrhoea and significant functional impairment and overall grade IV life threatening GvHD shortly after allo-HSCT. The patient failed to respond to methylprednisolone, cyclosporine A, infliximab, ruxolitinib and octreotide. The patient further received 4-time FMT treatment.<br>Donor 4 is a 25-year-old male and Donor is 48-year-old male. Both donor did not take any antibiotics or major immunosuppressive agents within the preceding 3 months and have no history of IBD, irritable bowel syndrome or chronic diarrhea, gastrointestinal malignancy or polyposis.
Recruitment	The patient was recruited in the Prince of Wales Hospital.<br>Donor was recruited by the Center for Gut Microbiota Research, The Chinese University of Hong Kong. To ensure donors were healthy and fit for stool donation, donors were first screened using a questionnaire followed by stool and blood tests to rule out any infectious disease such as HIV, hepatitis B or C, syphilis or any communicable diseases such as inflammatory bowel disease, irritable bowel syndrome, or gastrointestinal malignancy.<br>No potential recruitment biases were detected.
Ethics oversight	This study was approved by Joint Chinese University of Hong Kong-New Territories East Cluster Clinical Research Ethics Committee (The Joint CUHK-NTEC CREC, CREC Ref. No.: 2017.260 and 2018.443). The patient and his guardians have consented to participate in this study and agreed for publication of the research results.

Note that full information on the approval of the study protocol must also be provided in the manuscript.

Clinical data

Policy information about clinical studies

All manuscripts should comply with the ICMJEguidelines for publication of clinical research and a completedCONSORT checklist must be included with all submissions.

Clinical trial registration	Safety and Efficacy of Fecal Microbiota Transplantation (ClinicalTrials.gov Identifier: NCT04014413)
Study protocol	The full study protocol is available upon to the corresponding author.
Data collection	The clinical data was collected at the Center for Gut Microbiota Research, The Chinese University of Hong Kong from January 2018 to July 2018.
Outcomes	Primary outcome:<br>Reduction of the cumulative incidence of grade II-IV acute GvHD by day after serial FMTs.<br>Secondary outcome:<br>There are no adverse events and recurrence of acute GvHD.
